# Sleep Disturbance is Associated With Higher Plasma Aβ Levels in Cognitively Normal Adults—A Population-Based Cross-Sectional Study

**DOI:** 10.3389/fnagi.2020.615838

**Published:** 2021-01-18

**Authors:** Yao Gao, Shan Wei, Fan Gao, Ling Gao, Liangjun Dang, Suhang Shang, Chen Chen, Kang Huo, Jingyi Wang, Jin Wang, Qiumin Qu

**Affiliations:** ^1^Department of Neurology, The First Affiliated Hospital of Xi'an Jiaotong University, Xi'an, China; ^2^Huyi Hospital of Traditional Chinese Medicine, Xi'an, China

**Keywords:** Alzheime's disease, risk factor, sleep disturbance, plasma amyloid-beta, biomaker

## Abstract

**Objective:** Growing evidence suggests that sleep disturbance is a risk factor for Alzheimer's disease (AD). Amyloid-β (Aβ) deposition in the brain is a main pathophysiology of AD. Considering that peripheral Aβ level is associated with brain Aβ deposition, the present study investigated the relationship between sleep disturbance and plasma Aβ levels.

**Methods:** This is a population-based cross-sectional study. A total of 1,459 participants from a village in the suburbs of Xi'an, China, were enrolled from January 3, 2017 to March 26, 2017. Sleep quality was assessed using the Pittsburgh Sleep Quality Index (PSQI), and a PSQI score of <5 points was considered as good sleep quality and a PSQI score of >10 points as poor sleep quality. Cognitive function was assessed with the Mini-Mental State Examination (MMSE). Fasting venous blood was taken in the morning, and the plasma Aβ levels were measured using ELISA. The relationships between plasma Aβ levels and sleep quality were analyzed using multiple linear regression.

**Results:** Among the participants, 231 had poor sleep quality (15.83%). The log-transformed Aβ_40_ level had significant differences among the different sleep groups (*F* = 3.216, *p* = 0.040). The log-transformed Aβ_40_ level was higher in the poor sleep quality group than that in the general sleep quality group [87.17 (73.42, 107.34) vs. 89.69 (74.81, 125.79) pg/ml, *p* = 0.016]. In bivariate analysis, sleep quality was negatively associated with the log-transformed plasma Aβ_40_ level (β = −0.025, *p* = 0.011).

**Conclusion:** In the community population, poorer sleep quality is associated with a higher plasma Aβ_40_ level. This indicated that sleep disturbance might also involve in dysfunction of peripheral Aβ clearance.

## Introduction

Alzheimer's disease (AD) is the leading cause of dementia and is a growing public health crisis caused by cognitive impairment and a lack of effective treatment (Mayeux and Stern, 2012). Although the pathogenesis of AD is not fully clear, the presence of extracellular amyloid-β (Aβ) plaques and intracellular neurofibrillary tangles (NFTs) is the hallmark of AD pathology (Nedergaard, 2013; Tarasoff-Conway et al., 2015). Aβ, a 39–42 amino acid residue protein (Scheuner et al., 1996; Masters and Selkoe, 2012), is the main content of senile plaques. The excessive accumulation of toxic forms of Aβ in the brain had been hypothesized to result from an imbalance between its production and clearance (Masters and Selkoe, 2012). The researchers found that, in rodent models, enhanced peripheral Aβ clearance independently relieved the burden on the cerebral Aβ, suggesting a close relationship between the peripheral Aβ clearance and deposition of the cerebral Aβ (Xiang et al., 2015).

Sleep is an important physiological process (Ju et al., 2013, 2014), and previous research has demonstrated its benefits by removing a variety of potentially toxic waste products, including Aβ (Xie et al., 2013). Epidemiological studies have shown that up to 25–40% of patients with AD suffer from sleep disorders (Ju et al., 2014). Abnormal sleep patterns were found in patients with mild cognitive impairment (Ju et al., 2014). In healthy humans, imaging studies have revealed an association between shorter or poorer self-reported sleep duration and a higher burden of Aβ in the brain (Spira et al., 2013; Brown et al., 2016). In this regard, there is increasing evidence that sleep disorders may cause AD (Sprecher et al., 2015), partly by promoting the accumulation of Aβ in the brain (Spira and Gottesman, 2017). In rodents, it has been shown that the clearance of brain Aβ mainly occurs during sleep (Kang et al., 2009) through the lymphatic pathway (Xie et al., 2013; Lee et al., 2015), which operates most efficiently during sleep (Iliff et al., 2012). Clinical studies have also shown that the cerebrospinal fluid Aβ levels are the highest before sleep and the lowest after awakening (Ooms et al., 2014).

However, the relationships between sleep disturbance and peripheral Aβ levels are not clear. In this population-based cross-sectional study, we investigated the relationship between sleep and plasma Aβ in middle-aged and older adults in China. We hypothesized that poorer sleep quality is associated with higher plasma Aβ level.

## Materials and Methods

### Participants

From January 3, 2017 to March 26, 2017, the cluster sampling method was used to register villagers over 40 years old in the Qubao village near Xi'an. The living styles and demographics are similar between the village and other rural areas in Xi'an. The inclusion criteria included: (1) age 40 or above; (2) having resided in the Qubao village for over 3 years; (3) agreeing to participate in the study and fill in the questionnaire; (4) agreeing to the collection of the venous blood. Exclusion criteria were as follows: (1) taking sleeping pills for more than three times a week in the last 1 month; (2) without the required Pittsburgh Sleep Quality Index (PSQI) score; (3) with severe liver, kidney, thyroid, and hematopoietic system diseases; (4) with aberrant plasma Aβ_42_, Aβ_40_ levels (above the mean of 3 SDs); (5) with cognitive impairment; (6) with hemolysis in the sample; and (7) without apolipoprotein E (ApoE) genotype.

This study was approved by the First Affiliated Hospital of Xi'an Jiaotong University. Written informed consent was obtained from all participants.

### The Questionnaire Survey and Cognitive Assessment

All subjects were surveyed using a uniform questionnaire, with general information collected through a face-to-face consultation, followed by a physical examination and a blood sample collection. All questionnaire surveys and cognitive assessments were performed by neurologists, students, or nurses who had received a uniform training before the study.

The Mini-Mental State Examination (MMSE) was used to assess global cognitive function (Katzman et al., 1988). The cutoff values are as follows: ≤ 17 for illiteracy, ≤ 20 for primary school ( ≤ 6 years of education), and ≤ 24 for junior high school or above (>7 years of education). An MMSE score below the cutoff value was defined as cognitive impairment.

### Sleep Quality Assessment

The PSQI was used to assess the sleep quality over a month by face-to-face questionnaire (Chen et al., 2020). According to the PSQI score, sleep quality was divided into three different groups, scores of <5 for good sleep quality, scores of 6–10 for general sleep quality, and scores of > 10 for poor sleep quality.

### Laboratory Evaluation

After filling in the questionnaire, 10 ml of blood was extracted from the elbow vein of each participant after fasting for over 8 h, and the blood was put into the purple ethylenediaminetetraacetic acid (EDTA) anticoagulant tube and the red non-coagulant tube. The red tube of blood samples was sent to the biochemistry laboratory of the First Affiliated Hospital of Xi'an Jiaotong University for biochemical assessment [HDL-c, LDL-c, TG, TC, and fasting blood glucose (FBG)]. After the collection of blood samples from the purple tube, the samples were centrifuged at a speed of 3,000 revolutions per second (10 min) within 2 h. All samples were stored in a refrigerator at −80°C for further analysis. Plasma levels of Aβ_40_ and Aβ_42_ were determined using commercially available quantitative ELISA kits (Yuanye Co. Shanghai, China) with a sensitivity of 1.0 pg/ml for each assay. All measurements are in duplicate and the results are averaged. According to the manufacturer's protocol, genomic DNA in the blood samples from the EDTA anticoagulant tubes was extracted using the blood genomic DNA extraction kit [Tiangen Biotech Co., Beijing, China]. We amplified the ApoE gene fragment of 244 base pairs, including two polymorphic sites at amino acid residues 112 and 158, by the PCR thermocycler. The Sanger sequencing method (Sangon Co. Shanghai, China) was used to detect the sequence of PCR products. Finally, we used the direct interpretation of the sequencing chromatography to determine the ApoE genotype, and participants were classified as ApoEε4 non-carriers (E2/2, E2/3, and E3/3) and ApoEε4 carriers (E2/4, E3/4, and E4/4).

### Statistical Analysis

All data were analyzed using SPSS 25.0 software (IBM, Chicago, IL, USA), and *p* < 0.05 (double-tailed) was statistically significant. First, we tested the distribution of each covariate using the skewness, kurtosis, and p-p plots. The covariates that almost matched the normal distribution included age, pulse rate, body mass index (BMI), FBG, mean arterial pressure (MAP), total cholesterol (TC), total triglycerides (TG), LDL cholesterol (LDL-c), HDL cholesterol (HDL-c), log-transformed plasma Aβ_40_, log-transformed plasma Aβ_42_, and log-transformed plasma Aβ_42_/Aβ_40_ ratio, expressed as mean (SD), and compared among different groups using the one-way ANOVA test. The non-normal distribution covariates include education years expressed as median (interquartile range) and compared by the Kruskal–Wallis test. The categorical variables were expressed as numbers (percentage) and were compared by the Chi-square test.

Before comparing, all variances were tested for normality and homogeneity. As plasma Aβ_40_, Aβ_42_, and Aβ_42_/Aβ_40_ ratios are non-normal distribution, they were log-transformed for matching the normal distribution. If they were statistically significant, a pairwise comparison was carried out among the three groups using *post- hoc* tests. Multiple linear regression was used to investigate statistical significance after adjusting for other confounding factors, including age, sex, education years, smoking, drinking, history of hypertension, history of heart disease, history of cerebrovascular disease, BMI, FBG, TC, TG, LDL-c, and HDL-c. Partial correlation analysis was used to study the correlation between the PSQI score and the plasma Aβ level. Covariates included age, sex, education years, smoking, alcohol consumption, hypertension, heart disease, cerebrovascular disease, BMI, FBG, and blood lipids.

## Results

### The Demographics of the Participants

**Figure 1:**
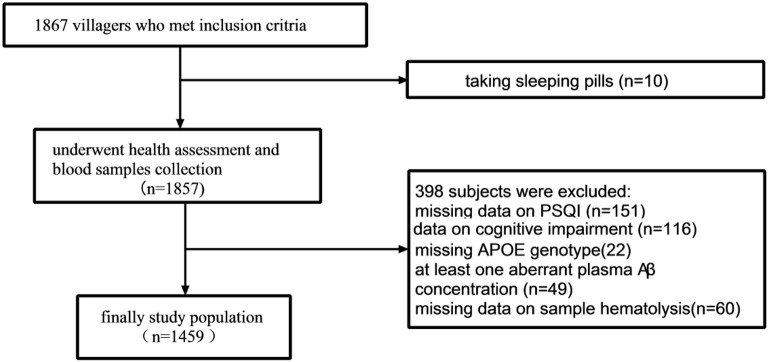
Flow chart of participant screening. Aβ, amyloid beta.

The study population screening process is shown in Figure 1. A total of 1,867 residents were investigated from January 3, 2017 to March 26, 2017. Hundred and fifty-one participants did not complete the PSQI questionnaire; 10 took sleeping pills, 49 had an aberrant plasma Aβ_42_ or Aβ_40_ level, 138 had missing data on cognitive impairment, 22 had missing ApoE genotype, and 60 had hemolysis. Finally, 1,459 participants were included in the analysis.

From a total of 1,459 subjects aged 40–86 years (mean 57.4±9.7 years), including 598 men (41%), 579 people (40%) had good sleep, 649 people (44%) had general sleep, and 231 people (16%) had poor sleep. Table 1 shows the demographic characteristics of the study participants. There were significant differences in sex, age, education, smoking, hypertension, and heart disease among different sleep groups.

**Table 1:** Characteristics of the total study population.

**Characteristics**	**Total (*n =* 1,459)**	**Good sleep (*n* = 579)**	**General sleep (*n* = 649)**	**Poor sleep (*n* = 231)**	**P-value**
Age, years	57.4 (9.7)	55.6 (9.0)	58.0 (10.0)	60.3 (9.6)	<0.001
Male, *n* (%)	598 (41%)	271 (46.8%)	264 (40.6%)	63 (27.3%)	<0.001
Education, years	7.0 (4.9)	8.0 (6.9)	7.0 (4.9)	6.0 (2.8)	<0.001
Hypertendion, *n* (%)	375 (24.7%)	111 (19.2%)	184 (28.4%)	80 (34.6%)	<0.001
Diabetes mellitus, *n* (%)	171 (11.7%)	59 (10.2%)	83 (12.8%)	29 (12.6%)	0.247
Cardiovascular disease, *n* (%)	77 (5.3%)	16 (2.8%)	38 (5.9%)	23 (10%)	0.001
Cerebrovascular disease, *n* (%)	106 (7.3%)	31 (5.4%)	53 (8.2%)	22 (9.5%)	0.139
Smoking, *n* (%)	461 (31.6%)	207 (35.8%)	206 (31.7%)	48 (20.8%)	0.001
Drinking, *n* (%)	176 (12.1%)	77 (13.3%)	75 (11.6%)	24 (10.4%)	0.394
Lack of physical activity, *n* (%)	337 (19.8%)	11 (16.7%)	153 (20.5%)	74 (25.3%)	0.008
BMI (kg/m^2^)	25.3 (3.5)	25.3 (3.1)	25.2 (3.8)	25.3 (3.3)	0.402
Pulse rate	74.1 (10.4)	73.9 (10.1)	74.0 (10.9)	74.9 (9.7)	0.391
Mean artery pressure, mmHg	96.1 (12.0)	95.5 (11.7)	96.4 (11.8)	96.5 (13.2)	0.298
Fasting blood glucose, mmol/l	5.7 (1.5)	5.7 (1.8)	5.7 (1.4)	5.7 (1.3)	0.807
Total cholesteral, mmol/l	5.16 (1.06)	5.13 (0.99)	5.21 (1.10)	5.11 (1.13)	0.279
TG, mmol/l	1.60 (1.11)	1.61 (0.92)	1.56 (1.25)	1.68 (1.17)	0.369
Low-density lipoprotein, mmol/l	2.62 (0.74)	2.64 (0.81)	2.64 (0.68)	2.53 (0.66)	0.152
High-density lipoprotein, mmol/l	1.60 (0.38)	1.58 (0.36)	1.63 (0.42)	1.60 (0.34)	0.073
APOE ε4 carriers, *n* (%)	184(12.6%)	68 (11.8%)	80 (12.3%)	36 (29.2%)	0.322

*One-way ANOVA or the Kruskal–Wallis test was used to compare the difference between the approximately normally distributed continuous variables among different sleep groups. The Kruskal–Wallis test and the median (quartile) were used for the skew distributional data and the Chi-square test, and the percentage was used for categorical variables. Data are mean (SD), median (interquartile range), or number (percentage). SD, standard deviation; BMI, body mass index; TG, triglyceride*.

### Relationship Between Plasma Aβ Levels and Sleep in the Total Population

**Figure 2:**
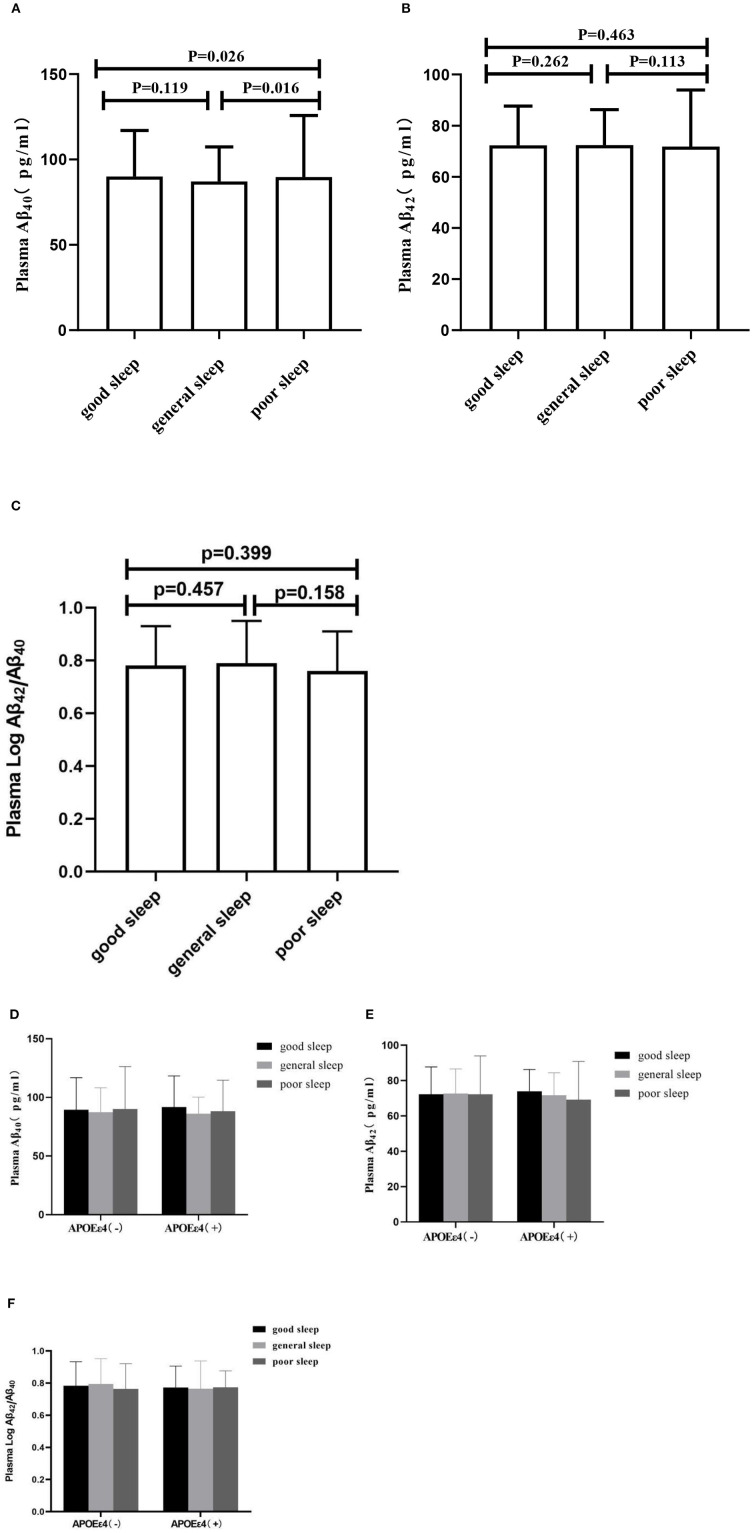
Plasma concentrations of Aβ_40_, Aβ_42_, and Aβ_42_/Aβ_40_. In **(A–C)**, the segments indicate the comparison between three sleep groups in total population. In **(D–F)**, the segments indicate the comparison in subgroups according ApoEε4 status. Aβ_40_, Aβ_42_, and Aβ_42_/Aβ_40_ matched the normal distribution after the log-transformed, the data are shown as median (interquartile range), indicated by dots and connecting lines.

As shown in Figure 2, the log-transformed plasma Aβ_40_ level had significant difference among different sleep groups (*F* = 3.216, *p* = 0.040). The Aβ_40_ level was higher in the poor sleep quality group than that in the general sleep quality group [87.17 (73.42, 107.34) vs. 89.69 (74.81, 125.79) pg/ml, *p* = 0.016]. There was no significant difference among different sleep groups (F = 1.437, *p* = 0.238) in log-transformed plasma Aβ_42_, nor was there any significant difference in log-transformed plasma Aβ_42_/Aβ_40_ ratio (F = 1.031, *p* = 0.357).

### Multivariate Analysis of the Relationship Between Plasma Aβ Levels and Sleep in the Total Population

In order to eliminate the influence of covariates, multiple linear regression analysis was performed. In the total population, after adjusting for age, gender, and education, sleep quality was negatively correlated with the log-transformed plasma Aβ_40_ level (β = −0.025, *p* = 0.013), and the PSQI score was positively correlated with the log-transformed plasma Aβ_40_ level (β = 0.009, *p* = 0.007) and negatively correlated with the plasma Aβ_42_/Aβ_40_ ratio level (Table 2, model 1). The log-transformed plasma Aβ_42_ had no significant correlation with sleep quality or the PSQI score. When other covariates (smoking, drinking, history of hypertension, history of heart disease, cerebrovascular disease, BMI, FBG, and blood lipids) were included in the multiple linear regression model, the results were approximately the same (Table 2, model 2).

**Table 2:** Multiple linear regression of sleep quality and plasma Aβ levels in total study participants (*n* = 1,459).

	**Aβ_40_**		**Aβ_42_**		**Aβ_42_/Aβ_40_**	
	**β**	***p***	**β**	***p***	**β**	***p***
**Model 1**						
Sleep quality	−0.025	0.013	−0.016	0.087	0.008	0.184
Sleep score	0.009	0.007	0.005	0.158	−0.005	0.048
**Model 2**						
Sleep quality	−0.025	0.011	−0.018	0.069	0.008	0.195
Sleep score	0.01	0.003	0.006	0.111	−0.005	0.040

*β, the unstandardized regression coefficient*.

*Model 1: adjust for sex, age, education years*.

*Model 2: adjust for age, sex, years of education, smoking, drinking, history of hypertension, history of heart disease, cerebrovascular disease, BMI, FBG, TC, TG, HDL-c, and LDL-c. BMI, body mass index; FBG, fast blood glucose; Aβ, amyloid-beta; TC, total cholesterol; TG, triglyceride; HDL-c, high-density lipoprotein; LDL-c, low-density lipoprotein*.

### Multivariate Analysis of the Correlation Between the Sleep Score and the Plasma Aβ Level

**Figure 3:**
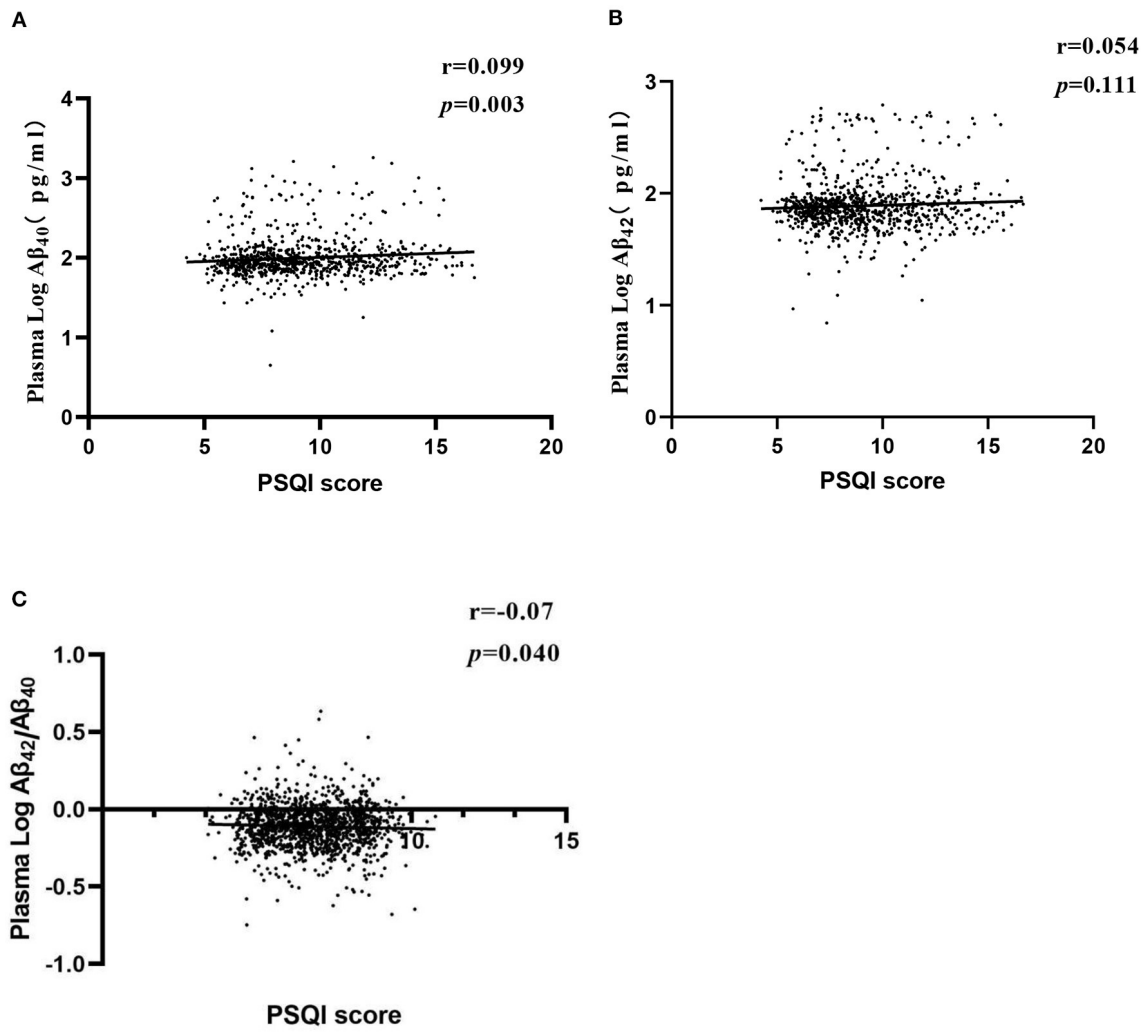
Partial correlations of PSQI score and plasma Aβ levels. Partial linear correlations between PSQI score and log-transformed Aβ_40_ level **(A)**, log-transformed Aβ_42_ level **(B)**, and log-transformed Aβ_42_/Aβ_40_ **(C)**. Adjusted for age, sex, years of education, smoking, drinking, history of hypertension, history of heart disease, cerebrovascular disease, BMI, FBG, TC, TG, HDL-c, and LDL-c. BMI, body mass index; FBG, fast blood glucose Aβ, amyloid beta; TC, total cholesterol; TG, HDL-c, triglyceride; HDL-c, high-density lipoprotein; LDL-c, low-density lipoprotein.

The partial correlation analysis was performed to investigate the correlation between the post-stroke cognitive impairment (PSCI) score and the plasma Aβ. After adjusting for age, sex, education, smoking, alcohol consumption, history of hypertension, history of heart disease, cerebrovascular disease, BMI, FBG, and blood lipid, the sleep score was positively correlated with the log-transformed plasma Aβ_40_ level (*r* = 0.099, *p* = 0.003) and negatively correlated with the plasma Aβ_42_/Aβ_40_ ratio level (*r* = −0.070, *p* = 0.040) but not with the log-transformed plasma Aβ_42_ (Figure 3).

### Effects of ApoEε4 Allele on Sleep Quality and Plasma Aβ Levels

ApoEε4 is the strongest genetic risk factor for AD (Corder et al., 1993; Bu, 2009) and had an influence on cognition and Aβ metabolism (Ellis et al., 1996; Huang and Mucke, 2012). A univariate analysis showed that plasma Aβ_40_ levels were higher in ApoEε4 carriers than in non-carriers [88.77 (74.11, 113.78) vs. 89.25 (74.08, 107.49) pg/ml, *p* = 0.020], but plasma Aβ_42_ levels did not show a significant difference between ApoEε4 carriers and non-carriers.

The PSQI scores had no significant difference between ApoEε4 carriers and non-carriers. Age, sex, education, smoking, alcohol consumption, history of hypertension, history of heart disease, cerebrovascular disease, BMI, FBG, and blood lipid had no significant differences between ApoEε4 carriers and non-carriers (Table 3).

**Table 3:** Difference of the plasma Aβ levels, sleep score, and other covariates between ApoEε4 carriers and non-carriers.

**Characteristics**	**APOEε4 non-carriers (*n =* 1,275)**	**APOEε4 carrier (*n =* 184)**	***P-*value**
Age, years	57.3 (9.9)	57.7 (8.7)	0.565
Male, *n* (%)	529 (41.6%)	69 (37.5%)	0.296
Education, years	7 (4.9)	7 (3.9)	0.141
Hypertendion, *n* (%)	334 (26.2%)	39 (21.2%)	0.200
Diabetes mellitus, *n* (%)	147 (11.5%)	24 (13.0%)	0.827
Cardiovascular disease, *n* (%)	69 (5.4%)	8 (4.3%)	0.467
Cerebrovascular disease, *n* (%)	90 (7.1%)	16 (8.7%)	0.680
Smoking, *n* (%)	406 (31.9%)	55 (29.9%)	0.798
Drinking, *n* (%)	158 (12.4%)	18 (9.8%)	0.471
Lack of physical activity, *n* (%)	233 (18.9%)	33 (18.3%)	0.283
BMI (kg/m^2^)	25.3 (3.7)	25.8 (4.8)	0.120
Pulse rate	74.3 (10.0)	74.1 (9.8)	0.797
Mean artery pressure, mmHg	95.3 (12.1)	96.5 (11.0)	0.773
Fasting blood glucose, mmol/l	5.7 (1.6)	5.8 (1.9)	0.381
Total cholesteral, mmol/l	5.18 (1.06)	5.03 (0.98)	0.069
TG, mmol/l	1.62 (1.16)	1.47 (0.73)	0.082
Low-density lipoprotein, mmol/l	2.64 (0.74)	2.53 (0.69)	0.084
High-density lipoprotein, mmol/l	1.60 (0.39)	1.60 (0.35)	0.915
Aβ40 (pg/ml)	88.77 (74.11, 113.78)	89.25 (74.08, 107.49)	0.020
Aβ42 (pg/ml)	72.29 (59.37, 88.03)	71.85 (55.66, 85.90)	0.121
Aβ42/Aβ40	0.79 (0.65,0.94)	0.77 (0.63, 0.91)	0.116
sleep score	6.75 (3.50)	7.08 (3.62)	0.236

*Unpaired Student's t-test and mean ± SD were used to compare the difference of the approximately normally distributed continuous variables between ApoEε4 carriers and non-carriers. The Mann–Whitney U-test and median (quartile) were used for the skew distributional data. The Chi-square and percentage were used for categorical variables. SD, standard deviation; BMI, body mass index; TG, triglyceride*.

### Stratified Multivariate Analysis of the Relationship Between Sleep Quality and Plasma Aβ According to ApoEε4 Status

Stratified binary logistic regression analysis, according to the ApoEε4 status, showed that sleep quality was negatively correlated with log-transformed plasma Aβ_40_ (β = −0.024, *p* = 0.010; Table 4, model 4) in ApoEε4 non-carriers but not in ApoEε4 carriers (Table 4, model 6). For Aβ_42_, there was no significant correlation between sleep quality in ApoEε4 carriers and non-carriers (Table 4, models 4 and 6). The ApoE genotypes are summarized in Table 5.

**Table 4:** Multiple linear regression of sleep quality and plasma Aβ levels in the subgroups according to the ApoEε4 status.

	**Aβ_40_**		**Aβ_42_**		**Aβ_42_/Aβ_40_**	
	**β**	***p***	**β**	***p***	**β**	***p***
**Model 3**						
Sleep quality	−0.023	0.033	−0.014	0.173	0.008	0.249
Sleep score	0.009	0.014	0.004	0.324	−0.05	0.055
**Model 4**						
Sleep quality	−0.024	0.027	−0.015	0.14	0.007	0.264
Sleep score	0.01	0.007	0.006	0.228	−0.005	0.045
**Model 5**						
Sleep quality	−0.033	0.136	−0.03	0.222	0.011	0.509
Sleep score	0.01	0.204	0.012	0.176	−0.003	0.628
**Model 6**						
Sleep quality	−0.03	0.177	−0.032	0.217	0.007	0.667
Sleep score	0.009	0.24	0.012	0.208	−0.003	0.555

*Models 3 and 4 were analyzed in ApoEε4 non-carriers. Models 5 and 6 were analyzed in ApoEε4 carriers*.

*β, the unstandardized regression coefficient*.

*Model 3 and model 5: adjust for sex, age, and education years*.

*Model 4 and model 6: adjust for age, sex, years of education, smoking, drinking, history of hypertension, history of heart disease, cerebrovascular disease, BMI, FBG, TC, TG, HDL-c, and LDL-c. BMI, body mass index; FBG, fast blood glucose Aβ, amyloid-beta; TC, total cholesterol; TG, triglyceride; HDL-c, high-density lipoprotein; LDL-c, low-density lipoprotein*.

**Table 5:** Frequency of ApoE genotypes in all cases.

**Genotype**	
E3/E3	1,054 (72.2)
E3/E4	160 (11.0)
E2/E3	221 (15.2)
E2/E4	12 (0.8)
E2/E2	–
E4/E4	12 (0.8)

*Data are presented as n (%)*.

**Figure 4:**
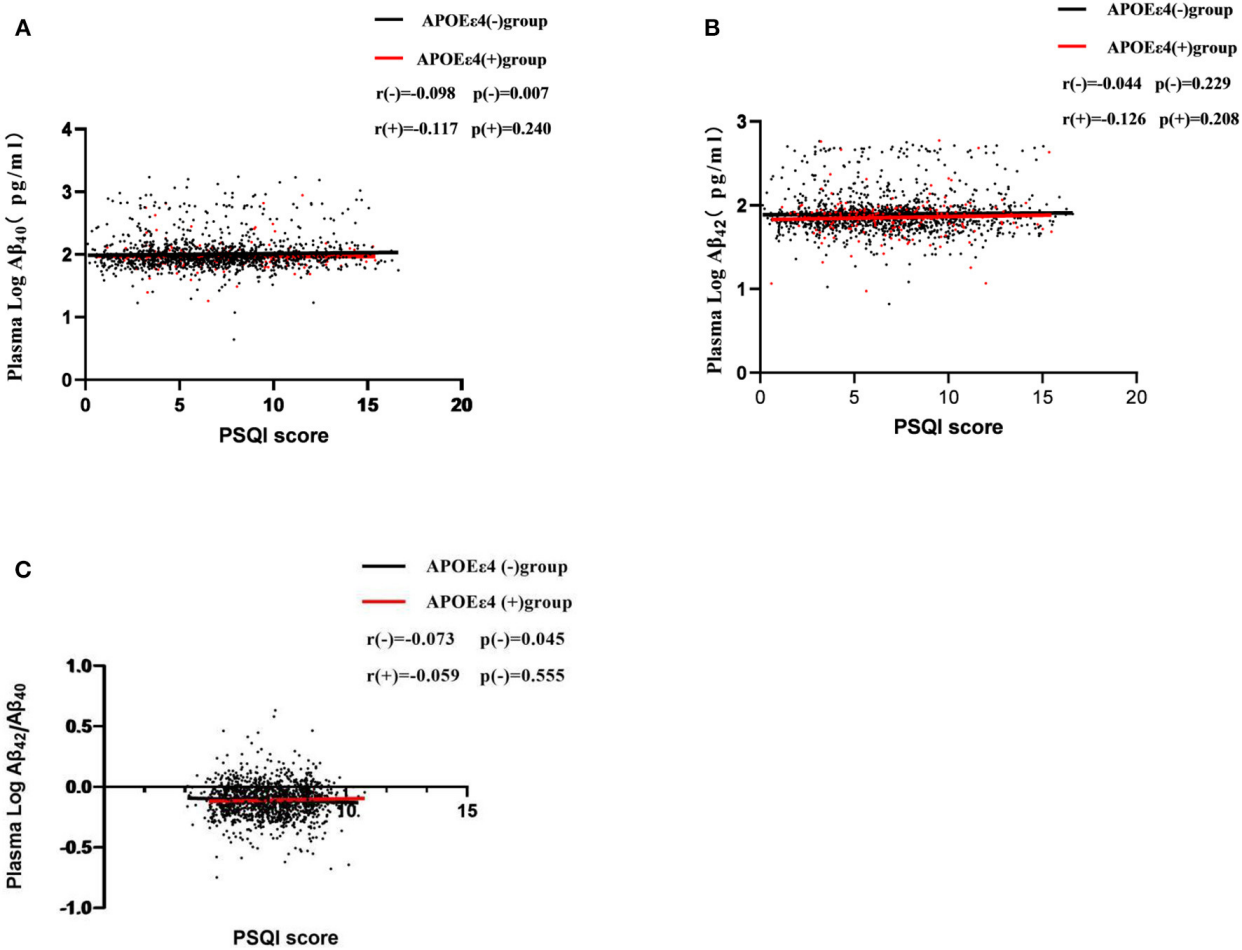
Partial correlations of sleep scores (PSQI score) and plasma Aβ levels according APOE ε4 status. Participants were divided into two groups: APOE ε4 carriers and non-carriers, which are represented by red dots and black dots.

The stratified partial correlation analysis showed that after adjusting for age, gender, years of education, smoking, alcohol consumption, history of hypertension, history of heart disease, cerebrovascular disease, BMI, FBG, and blood lipid, the sleep score was positively correlated with the log-transformed plasma Aβ_40_ (*r* = 0.098, *p* = 0.007) and negatively correlated with the plasma Aβ_42_/Aβ_40_ ratio (*r* = −0.073, *p* = 0.045) in ApoEε4 non-carriers but not in ApoEε4 carriers. There was no correlation between the PSQI score and the plasma Aβ_42_ levels neither in the ApoEε4 carriers nor in the non-carriers (Figure 4).

## Discussion

In this population-based study, we found that sleep quality was negatively correlated with the plasma Aβ_40_ levels and the plasma Aβ_42_/Aβ_40_ ratio and was positively correlated with the PSQI score and the plasma Aβ_40_ levels. There was no correlation between sleep quality/the PSQI score and the plasma Aβ_42_ levels. These findings were independent of age, sex, and other confounding factors.

More and more evidence have demonstrated that sleep disturbance is a risk factor for AD (Ju et al., 2013, 2014) and promoted Aβ accumulation in the brain (Ju et al., 2017). Although peripheral Aβ is closely associated with Aβ deposition in the brain (Roberts et al., 2014), the relationship between sleep disturbance and plasma Aβ is not clear. In the present study, we found that the plasma Aβ_40_ level was higher in the poor sleep group, and the multiple linear regression analysis showed that the worse the sleep quality, the higher the plasma Aβ_40_ level. In addition, we found that the worse the sleep quality, the lower the plasma level of Aβ_42_/Aβ_40_ ratio, which may be due to the decrease of plasma Aβ42 level or the increase of plasma Aβ40 level or both of them. These suggested that sleep disturbance was associated with the plasma Aβ_40_ level and the plasma Aβ_42_/Aβ_40_ ratio level.

In this large samples community population-based study, the participants were chosen by random cluster sampling from selected villages, and the population composition was similar to that in the rural areas of Xi'an. The sleep quality of all participants was evaluated face-to-face using PSQI. Although PSQI is a self-rated questionnaire for sleep disturbance, it has been demonstrated that PQSI is a reliable and effective measure of insomnia, with a diagnostic sensitivity of 89.6% and specificity of 86.5% for sleep disturbance (Buysse et al., 1989). These assure that the results actually represent the relationship between sleep disturbance and the plasma Aβ level.

The mechanism of sleep disturbance related to plasma Aβ levels is unclear. In our recent study, we found that chronic sleep restriction led to an increase of cortical Aβ oligomers levels in the rats (Rothman et al., 2013), which may be due to an upregulation of β-secretase (Zhao et al., 2017) and Aβ clearance dysfunction by transporters lipoprotein receptor-related protein-1 (LRP1) and a receptor for advanced glycation end products (RAGE). Meanwhile, the soluble form of LRP1 (sLRP1) and the soluble form of RAGE (sRAGE) in the plasma were significantly reduced after sleep deprivation (Zlokovic et al., 2010), which might also lead to Aβ_40_ peripheral clearance dysfunction (Sehgal et al., 2012). Oxidative stress induced by sleep deprivation may destroy the binding of Aβ with sLRP1 and sRAGE, leading to the disruption of peripheral clearance of Aβ (Cai et al., 2016; Wei et al., 2017).

In the present study, we did not find any relationship between sleep disturbance and the plasma Aβ_42_ level. Compared with Aβ_40_, Aβ_42_ is more cytotoxic but of the less common Aβ type. In fact, the Aβ_42_ level in the plasma may be a less sensitive biomarker compartment than that in the cerebrospinal fluid. It might not be due to the dilution of peripheral blood, but the presence of erythrocyte membrane proteins, such as albumin, as well as multiple other circulating Aβ-binding molecules altered the measurable plasma levels of Aβ in the periphery (Metti et al., 2013; Ooms et al., 2014). These might mask the relationships between sleep disturbance and Aβ_42_ concentration.

The relationship between sleep and plasma Aβ levels were not only found in the ApoEε4 carriers but also in the non-carriers. The reasons are not clear. The PQSI score had no significant difference between the ApoEε4 carriers and the non-carriers, suggesting that ApoE has no isoform-specific effect on human sleep. As all know, ApoEε4 is the strongest genetic risk factor for AD (Corder et al., 1993; Bu, 2009), and it accelerates Aβ accumulation in the brain (Marques et al., 2009; Huang and Mucke, 2012). Studies in human and transgenic mice have shown that the Aβ levels in the brain and the amyloid plaque load are ApoE isoform-dependent (ε4>ε3>ε2) (Reiman et al., 2009), suggesting that ApoE plays an important role in the regulation of Aβ metabolism, aggregation, and deposition (Bales et al., 2009). In the present study, we found that the plasma Aβ_40_ level was higher in the ApoEε4 carriers than that in the non-carriers, suggesting that ApoE had also an isoform-specific effect on human plasma Aβ_40_ transport, which may mask the relationship between plasma Aβ levels and sleep quality in the ApoE 4 carrier.

There are some limitations. First, the design of the cross-sectional study does not allow for a causal hypothesis between plasma Aβ levels and sleep disturbance. It is difficult to determine whether sleep disorders cause plasma Aβ changes because Aβ deposition in the brain and sleep disturbance are considered to be bidirectional (Ju et al., 2013, 2014), as an increase of the burden of Aβ in the brain may also contribute to sleep disturbances. These need to be validated in additional longitudinal cohort studies. Second, deposits of Aβ in the brain or cerebrospinal fluid cannot be obtained. It is difficult to determine whether the increase of peripheral Aβ accompanies Aβ accumulation in the brain. Third, as the PSQI is a subjective measurement of sleep quality, we did not use any sleep monitoring devices, such as polysomnography and activity tracing, to assess the sleep condition objectively, which may cause recall bias.

## Conclusion

In summary, the present study shows that, in community populations, poorer sleep quality is associated with the higher plasma Aβ_40_ level and the lower plasma Aβ_42_/Aβ_40_ ratio level. This indicated that sleep disturbance might also be involved in peripheral Aβ clearance dysfunction. However, the underlying mechanism is not clear. Additional large sample cohort studies are needed.

## Data Availability Statement

The raw data supporting the conclusions of this article will be made available by the authors, without undue reservation.

## Ethics Statement

The studies involving human participants were reviewed and approved by Medical Ethics Committee of the First Affiliated Hospital of Xi'an Jiaotong University. The patients/participants provided their written informed consent to participate in this study.

## Author Contributions

YG participated in the questionnaire survey and biochemical assessment, conducted the results analysis, and wrote the manuscript. FG, LG, and SW participated in the questionnaire survey, sample collection, and biochemical assessment. SS designed this study and participated in the questionnaire survey and sample collection. CC, LD, JinW, KH, and JingW participated in the questionnaire survey and sample collection. QQ coordinated and supervised all stages of the project. All authors have read and approved the final version of the manuscript.

## Conflict of Interest

The authors declare that the research was conducted in the absence of any commercial or financial relationships that could be construed as a potential conflict of interest.
